# Observation of the cervical microbiome in the progression of cervical intraepithelial neoplasia

**DOI:** 10.1186/s12885-022-09452-0

**Published:** 2022-04-04

**Authors:** He Wang, Yanming Jiang, Yuejuan Liang, Lingjia Wei, Wei Zhang, Li Li

**Affiliations:** 1 Department of gynecologic oncology, Guangxi Medical University Cancer Hospital, 71 He Di Road, Nanning, 530021 Guangxi China; 2 Department of Obstetrics and Gynecology, People’s Hospital of Guangxi Zhuang Autonomous Region, Nanning, China; 3 Department of Obstetrics and Gynecology, Liuzhou People’s Hospital, Liuzhou, China; 4 Department of Obstetrics and Gynecology, Guangxi Medical University, Nanning, China

**Keywords:** Cervical intraepithelial neoplasia (CIN), cervical microbial community, malignant transformation, prediction model

## Abstract

**Objective:**

Cervical microbial community in the cervical intraepithelial neoplasia and cervical cancer patients was analysed to study its composition, diversity and signalling pathways by high-throughput 16S rDNA sequencing,and the candidate genes associated with occurrence and progression of cervical intraepithelial neoplasia were screened out and the model was established to predict the evolution of cervical intraepithelial neoplasia malignant transformation from the cervical microbial genes aspect.

**Methods:**

Cervical tissues of normal, cervical intraepithelial neoplasia and cervical cancer patients without receiving any treatment were collected. The correlation between candidate genes and cervical intraepithelial neoplasia progression was initially determined by analyzing the microbial flora. Real-time fluorescence quantitative PCR was used to detect the expression of candidate genes in different cervical tissues, ROC curve and logistic regression was used to analyse and predict the risk factors related to the occurrence and progression of cervical intraepithelial neoplasia. Finally, the early warning model of cervical intraepithelial neoplasia occurrence and progression is established.

**Results:**

Cervical tissues from normal, cervical intraepithelial neoplasia and cervical cancer patients were collected for microbial community high-throughput 16S rDNA sequencing. The analysis revealed five different pathways related to cervical intraepithelial neoplasia. 10 candidate genes were selected by further bioinformatics analysis and preliminary screening. Real time PCR, ROC curve and Logistic regression analysis showed that human papillomavirus infection, TCT severity, ABCG2, TDG, PCNA were independent risk factors for cervical intraepithelial neoplasia. We used these indicators to establish a random forest model. Seven models were built through different combinations. The model 4 (ABCG2 + PCNA + TDG) was the best early warning model for the occurrence and progression of CIN.

**Conclusions:**

A total of 5 differential pathways and 10 candidate genes related to occurrence and progression of cervical intraepithelial neoplasia were found in cervical microbial community. This study firstly identified the genes from cervical microbial community that play an important role in the occurrence and progression of cervical intraepithelial neoplasia. At the same time, the early warning model including ABCG2 + PCNA+TDG genes provided a new idea and target for clinical prediction and blocking the evolution of cervical intraepithelial neoplasia malignant transformation from the aspect of cervical microbiological related genes.

## Background

Cervical intraepithelial neoplasia (CIN) is the precancerous lesion of cervical cancer. A lot of studies have demonstrated that human papilloma virus (HPV) is the main carcinogen responsible for CIN and cervical cancer. However, some other studies found that not all patients infected with HPV must become CIN or cervical cancer [1]. Although it is now believed that adjuvant factors other than HPV play a key role in the development of cancer, most of the potential mechanisms of this carcinogenic effect are still unknown [2]. It has been shown that the cause of human diseases is not only a single pathogen, but also involves the overall changes in the human microbiology group [3]. In recent years, with the application of metagenomic principles and the development of high-throughput sequencing analysis, research on the relationship between microorganisms and human diseases has been initiated. At present, the most common sequencing methods to identify the microbiome are pyrosequencing and 16S rRNA sequencing. MitraA [4] recently performed 16S rRNA gene amplification of the vaginal wall microorganisms of 52 cases of LSIL, 92 cases of HSIL, 5 cases of ICC, and 20 normal controls. The results indicated that vaginal microbial diversity is associated with the severity of CIN disease and that microbes can participate in regulating the persistence of viral infections and disease progression. The role of Cervical microbial community in the progression of CIN has gradually been recognized, and its synergy with HPV in CIN and cervical cancer is expected to become a hot spot in cervical disease research. Through random forest model, vaginal microbiome-derived bacterial markers can be used as a predictive model to predict the CIN malignant transformation, indicates that vaginal microbiome may play a role as biomarker. Current research on the correlation between CIN and vaginal microbes is mostly about the composition of the vaginal microbial flora and the pathogenic mechanism of the bacterial species, the severity of the bacterial flora and CIN, the relationship between the changes of the CIN flora and cervical cancer, and there are no studies involving genes and pathways. And no researchers have focused on the role of cervical tissue microecology and its related genes in the progression of CIN. Therefore, we analysed the cervical microbial community by high-throughput 16S rDNA sequencing, bioinformatics analysis and real time PCR to study its composition, diversity and signalling pathways in patients with CIN and cervical cancer. Finally, we screened out the candidate genes associated with occurrence and progression of CIN and established the best model to predict the evolution of CIN malignant transformation. Through this study, we proposed the important role of cervical microbial community and its related genes in the process of cervical cells carcinogenesis which is never discovered before**.**

## Materials and Methods

### Selection of study cases

Thirty-eight cases of CIN tissues (9, 11 and 18 cases were CIN1, 2 and 3 respectively) were randomly selected in Affiliated Cancer Hospital of Guangxi Medical University from May 2015 to July 2015; 14 cases of normal cervical tissue (taken from patients with uterine fibroids requiring hysterectomy) and 10 cervical cancer tissues were subjected to high-throughput 16S rDNA sequencing of microbial communities, and a series of analyses were performed. Fifty-two cases of CIN tissue, 38 cases of normal cervical tissue and 30 cases of cervical cancer tissue from January 2017 to December 2018 were again selected for realtimefluorescence quantitative PCR detection. All cases were discovered for the first time and confirmed by histopathology. No treatment was performed before the operation, and human papillomavirus detection and cervical cytology were performed before treatment. No trichomonas, Candida infection or bacterial vaginosis was detected in the vaginal secretions within 3 days before sampling. Subjects were required to abstain from sexual intercourse 3 days before sampling. No drugs affecting the vaginal flora were used before sampling.

### Cervical sample collection and method

Samples were placed in a sterile tube for cryopreservation and immediately stored in liquid nitrogen. After the samples were collected, they were transferred to the laboratory for storage at − 80 °C. For the isolation, extraction and purification of total bacterial DNA, mechanical (magnetic bead repeated beating method, Fast Prep FP120) combined with enzymatic methods (QIAam DNA Mini Kit, QIAGEN, Valencia, CA, USA) were used to efficiently extract relevant microbial DNA. DNA samples are detected by fluorescence quantification and agarose gel electrophoresis. We collected 1 μL for fluorescence quantitative detection (instrument: QubitFluorometer,manufacturer: Thermo Fisher), and 5 μL DNA for electrophoresis detection (agarose gel electrophoresis utilizes 1% agarose gel electrophoresis at 150 V for approximately 40 min) of integrity and presence of RNA or protein and secondary metabolite contamination.

### 16SrDNA V4 region target fragment library construction

The total 30 ng of DNA was used as the template, and the V4 region of the bacterial 16S rDNA was used as the target. The universal primers fused with the Miseq platform sequencing platform were used for primer design and synthesis, and New England Biolabs’ Phusion High-Fidelity PCR Master Mix with GC Buffer was used. High-efficiency and high-fidelity enzymes were employed for PCR: bacterial 16S rDNAprimers: 515F(5′-GTGCCAGCMGCCGCGGTAA-3′)/806R(5′-GGACTACHVGGGTWTCTAAT-3′); fusion primer design: forward primer for fusion V4 region: adapter+bracode+GTGCCAGCMGCCGCGGTAA F; reverse primer for fusion V4 region: adapter+bracode+GGACTACHVGGGTWTCTAAT R. The PCR reaction system (50 μL) consisted of DNA (30 ng) PCR Primer Cocktail*(4 μL) PCR Master Mix (25 μL)H_2_O(as needed). The PCR amplification reaction conditions were as follows: 94 °C pre-denaturation for 3 min; 98 °C for 45 s, 55 °C for 45 s, and 72 °C for 45 s for 30 cycles; 72 °C extension for 7 min. Library fragment recovery, purification and detection were performed using magnetic beads to screen the target Amplicon fragments; an Agilent 2100 Bioanalyzer (reagent: Agilent DNA 1000 Kit, cat No.5067–1504, manufacturer: Agilent) was used to detect the range of insert fragments in the library. An ABI StepOnePlus Real-Time PCR System (TaqMan Probe) was used to quantify the concentration of the library. According to the results of the library test, the samples are mixed and loaded on the machine, and the sample amount of each library was calculated. All libraries were mixed at a ratio of 1:1, and after mixing, the samples are fully shaken and sequenced using the Miseq sequencing platform. The sequencing was commissioned by Huada Gene Corporation.

### Sequencing data processing

Data quality control was performed with QIIME software [5] to filter low-quality data, and the linker and primer sequence, polybase N, poly A/T tail and low-quality bases at the end of the sequence were removed offline to obtain high-quality data. Data splitting was performed using the barcode sequence to split the data into different sample data. The allowed number of mismatches between the barcode sequence and sequencing reads was 0 bp; the barcode identification sequence and PCR amplification primer sequence were cut off, and the number of sequences in each sample was statistically analysed. Tag splicing was performed with FLASH software [6] to splice a pair of overlapping sequences in each sample. The tags were truncated from the first low-quality base site where the number of consecutive low-quality values (default setting <=10) reached the set length (default of 5). Tags less than 70% of the average length of tags in the collection were filtered out after interception.

### Species classification and abundance and analysis of pathways related to CIN

#### Species classification analysis

The software USEARCH (v7.0.1090) [7] was used to cluster the spliced tags into OTUs, and the sequences were clustered into operational taxonomic units (OTUs) based on 97% similarity. With a similarity of 97%, the number of OTUs for each sample was obtained, and the Venn diagram was used to show the number of common and unique OTUs for multiple samples and to visually show the overlap of OTUs between samples. The species represented by OTUs were combined to identify the core microorganisms in different environments. According to the abundance file of each sample OTU in each sample, the OTU of each sample or group was calculated to generate a Venn diagram using the VennDiagram package in R (v3.0.3) language. The obtained OTU representative sequence was annotated to obtain the species classification.

#### Species abundance and pathway analysis related to CIN

PICRUSt software uses marker gene data and reference genome databases to predict the functional composition of metagenomics. This method was first published in Nature Biotechnology in 2013 by Langille et al. [8]. We used the PICRUSt software in the KEGG database: Kyoto Encyclopaedia of Genes and Genomes database (59.3) (http://www.genome.jp/kegg/). After normalization according to the different 16 s rDNA copy numbers contained in each species, the possible abundance of each species in each sample was obtained, and the abundance value of each function was multiplied by the abundance value of the species and the abundance value of its potential function. Next, we combined the respective abundance values of all functions in the same sample to convert them into relative abundance values. A non-parametric rank sum test on the KEGG classification data was performed, using *P* < 0.05 and a false discovery rate (FDR) < 0.3 to denote a difference. COREMINE was used for biological annotation and text mining of differential pathways to further illustrate the relationship between differential signalling pathways and CIN.

### Bioinformatics analysis of differential pathways, preliminary screening of candidate genes and biological annotation of their correlation with the progress of bacteria and CIN

The KEGG (https://www.kegg.jp/kegg/) database was again applied to query the genes contained in the five differential pathways. The search tool STRING online database was employed to construct a protein-protein interaction network diagram (PPI) for the genes of each pathway, Cytoscape was used to visualize the analytic results, and then MOCDE was applied to cluster the results according to the given network diagram to identify areas of dense connections. The selection criteria were as follows: MOCDE scores> 5 points, degree cut-off = 2, node score cut-off = 0.2, Max depth = 100 and k-score = 2. Combined with the PubMed literature search, 10 candidate differential genes were initially screened out. The correlation between candidate differential genes and the progress of bacteria and CIN was biologically annotated using the online software COREMINE (http://www.coremine.com/medical/).

### Expression verification and analysis of candidate genes

Tissue total RNA was extracted using the TRIzol method, and the total RNA concentration and OD value (260/280) were determined using the SYnergy multifunctional microplate fluorescence analyser. The total RNA reverse transcription kit and real-time fluorescent quantitative PCR kit SYBR Premix Ex Taq™ II (TliRNaseH Plus) were provided by Takara, Japan. The synthesis of primers was completed by Takara Corporation of Japan, using β-Actin (ACTB) as the internal reference gene. The primer sequences are shown in Table 1. The fluorescence quantitative PCR reaction conditions were as follows: 95 °C predenaturation for 5 min, 95 °C for 30 s, 1 cycle; 95 °C for5 s, 60 °C for 30 s, 40 cycles; and 60 °C for 30 s, 40 cycles. ROC curve analysis was used to predict the value of candidate genes for early warning signs of CIN occurrence and progression, and logistic regression was used to analyse the risk factors related to CIN occurrence and progression.

**Table 1:** Fluorescence quantitative PCR primer sequence

Gene name	Forward Primer(5′ - > 3′)	Reverse Primer(5′ - > 3′)
ATM	TTGATCTTGTGCCTTGGCTAC	TATGGTGTACGTTCCCCATGT
ABCG2	ACGAACGGATTAACAGGGTCA	CTCCAGACACACCACGGAT
PCNA	CCTGCTGGGATATTAGCTCCA	CAGCGGTAGGTGTCGAAGC
XRCC1	CCTTTGGCTTGAGTTTTGTACG	CCTCCTTCACACGGAACTGG
HMGB1	TATGGCAAAAGCGGACAAGG	CTTCGCAACATCACCAATGGA
OGG1	ACTCCCACTTCCAAGAGGTG	GGATGAGCCGAGGTCCAAAAG
LIG1	ACAGTTCCCCATCAGGGATTC	CTCTGTGAGGCTTTCTTTCGG
SMUG1	GAGGAGCTTCGGCTCAATG	CGAGTCACGTAGTTGCGATG
FEN1	CACCTGATGGGCATGTTCTAC	CTCGCCTGACTTGAGCTGT
TDG	TGAAGCTCCTAATATGGCAGTTG	TTCCACTGGTTGTTTTGGTTCT
ACTB	CATGTACGTTGCTATCCAGGC	CTCCTTAATGTCACGCACGAT

## Results

### Analysis of clinical data of samples

Among the 62 samples, the oldest was 62 years old and the youngest was 22 years old. The average age was 33 ± 5.12 years. There was no significant difference in age between groups (*P* > 0.05), and there were no significant differences in parity or contraceptive methods (Table 2) (*P* > 0.05). The infection rate of high-risk HPV (hrHPV) also gradually increased with the aggravation of cervical lesions (*P* < 0.05).

**Table 2:** Analysis of clinical data of samples

	Normal (*n* = 14)	CIN 1 (*n* = 9)	CIN 2 (*n* = 11)	CIN 3 (*n* = 18)	Cervical cancer (*n* = 10)	*P* value
Age	32 ± 5.15	33 ± 4.23	33 ± 4.74	34 ± 5.68	34 ± 5.68	0.702
Contraceptive methods						0.997
IUD	5	4	4	8	4	
Condom	5	3	4	6	3	
OTC	2	2	3	3	3	
Parity						0.756
≥ 2	3	4	4	5	4	
< 2	11	5	7	13	6	
hrHPV						**0.000**
Negative	13	6	2	3	2	
Positive	1	3	9	15	8	

### Sequencing data and OTU construction

**Fig. 1:**
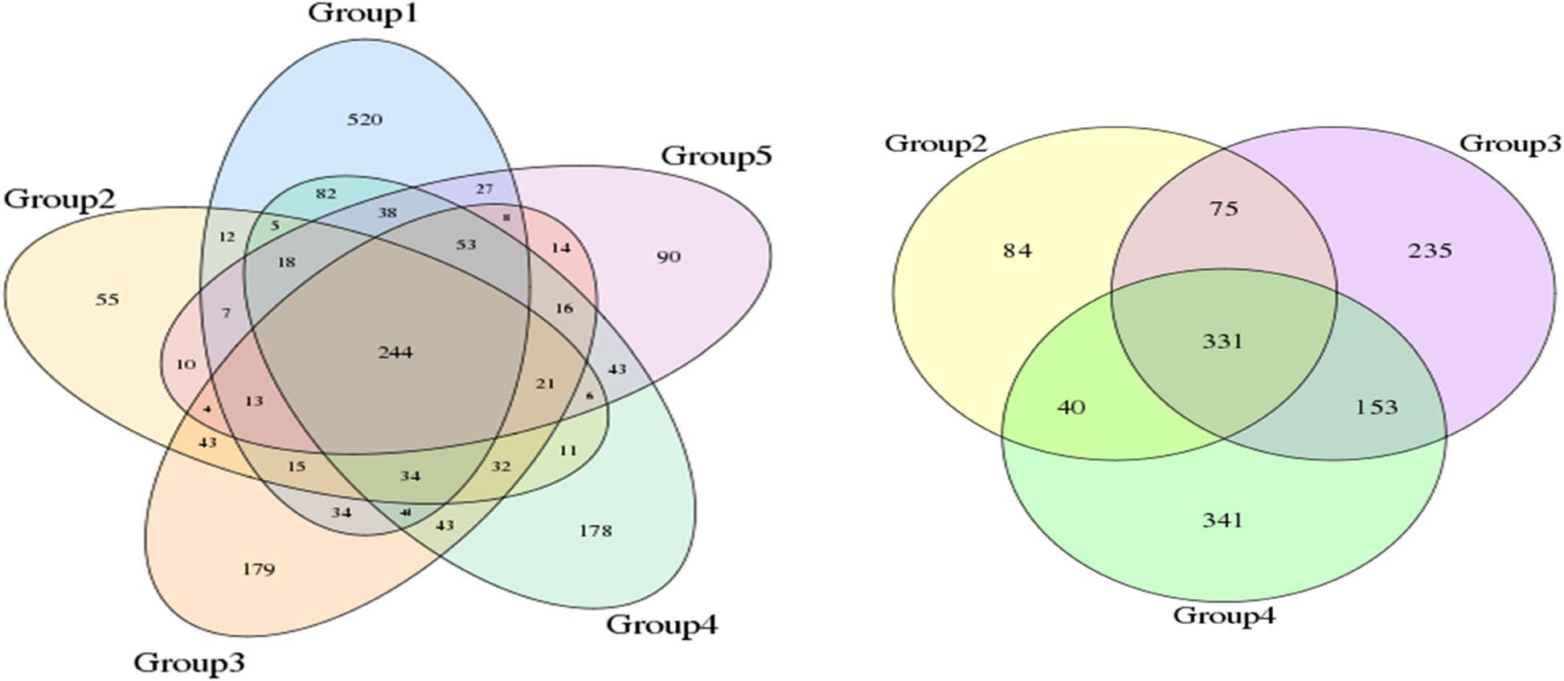
OTU venn diagram (Group 1, Normal group; Group 2, CIN1 group; Group 3, CIN2 group; Group 4, CIN3 group; Group 5, Cervical cancer group)

A total of 60 samples in 5 groups of specimens were analysed by high-throughput sequencing (2 samples were unqualified): Group 1 (normal group) 14 cases; Group 2 (CIN1 group) 9 cases; Group 3 (CIN2 group) 11 cases; Group 4 (CIN3 group) 17 cases; Group 5 (cervical cancer group) 9 cases. The 16S rDNA gene fragments were extracted from the samples, with the exception of low-quality sequences. The tags were spliced into tags through the overlap relationship between reads. A total of 1,873,442 tags were obtained for all samples, with an average of 31,224 tags per sample; the average tag length was 253 bp, and the SD value was 7 bp. The statistical results for the tags from each sample were as follows: A1-A14: normal group; B1-B9: CIN1 group; C1-C11: CIN2 group; D1-D17: CIN3 group; E1-E9: cervical cancer group. A total of 1896 OTUs were generated from 60 samples. For each sample OTU, the shared and unique OTUs between samples or groups and the Venn diagram between samples or groups are shown in Fig. 1.

### Species classification analysis results

From the class level analysis, β-Proteobacteria, γ-Proteobacteria, Bacillus, α-Proteobacteria, and Clostridium were the dominant strains in the normal group, CIN group and cervical cancer group, and each group had different dominant strains, namely, the normal group consisted of δ-Proteobacteria, the CIN1 and CIN2 groups were Sphingomyces, and the CIN3 and cervical cancer groups were Bacteroides (Table 3).

**Table 3:** Class classification level strains

Normal		CIN1		CIN2		CIN3		Cervical cancer	
Classification	%	Classification	%	Classification	%	Classification	%	Classification	%
Betaproteobacteria	8	Betaproteobacteria	8	Betaproteobacteria	15.5	Betaproteobacteria	10	Betaproteobacteria	9
Gammaproteobacteria	46	Gammaproteobacteria	20	Gammaproteobacteria	35	Gammaproteobacteria	31	Gammaproteobacteria	38
Bacilli	12	Bacilli	40	Bacilli	9.5	Bacilli	25	Bacilli	6.5
Alphaproteobacteria	9.5	Alphaproteobacteria	8	Alphaproteobacteria	6	Alphaproteobacteria	8	Alphaproteobacteria	8
Clostridia	1.5	Clostridia	1	Clostridia	4	Clostridia	3.5	Clostridia	10
Deltaproteobacteria	1	Saprospirae	9	Saprospirae	10	Bacteroidia	6	Bacteroidia	12

At the subordinate level, the dominant strains of Lactobacillus accounted for a large proportion in each group, but some samples in the normal group still did not show the dominant Lactobacillus. The other main microorganisms in CIN1/2 were Stenotrophom, Chitinophaga, and Acinetobacter in CIN3 and in cervical cancer were Halomonas, Shewanella, and Acinetobacter (Table 4).

**Table 4:** Genus classification level strains

Normal		CIN1		CIN2		CIN3		Cervical cancer	
classification	%	classification	%	classification	%	classification	%	classification	%
Lactobacillus	11	Lactobacillus	40	Lactobacillus	8	Lactobacillus	20	Lactobacillus	0.5
Halomonas	27	Stenotrophomonas	9	Stenotrophomonas	10	Halomonas	13	Halomonas	20
Shewanella	10	Chitinophaga	8	Chitinophaga	12	Shewanella	10	Shewanella	8
Acinetobacter	3.5	Acinetobacter	1.5	Acinetobacter	1	Acinetobacter	2	Acinetobacter	3
Pseudomonas	0.5	Pseudomonas	2	Sediminibacterium	1.5	Stenotrophomonas	5	Pseudomonas	1
Stenotrophomonas	0.5	Sediminibacterium	1	Bacteroides	3.5	Chitinophaga	4	Prevotella	5

### Species abundance and pathway analysis using the CIN group flora

**Fig. 2:**
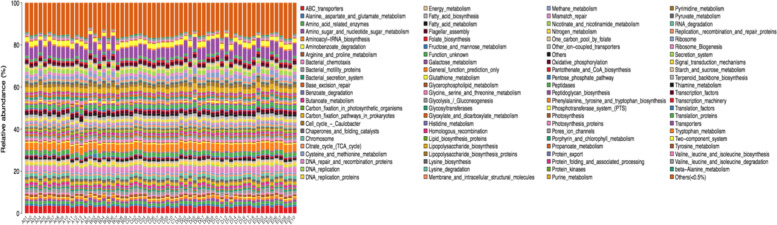
Analysis of pathways in which the flora contains genes mainly involved

**Fig. 3:**
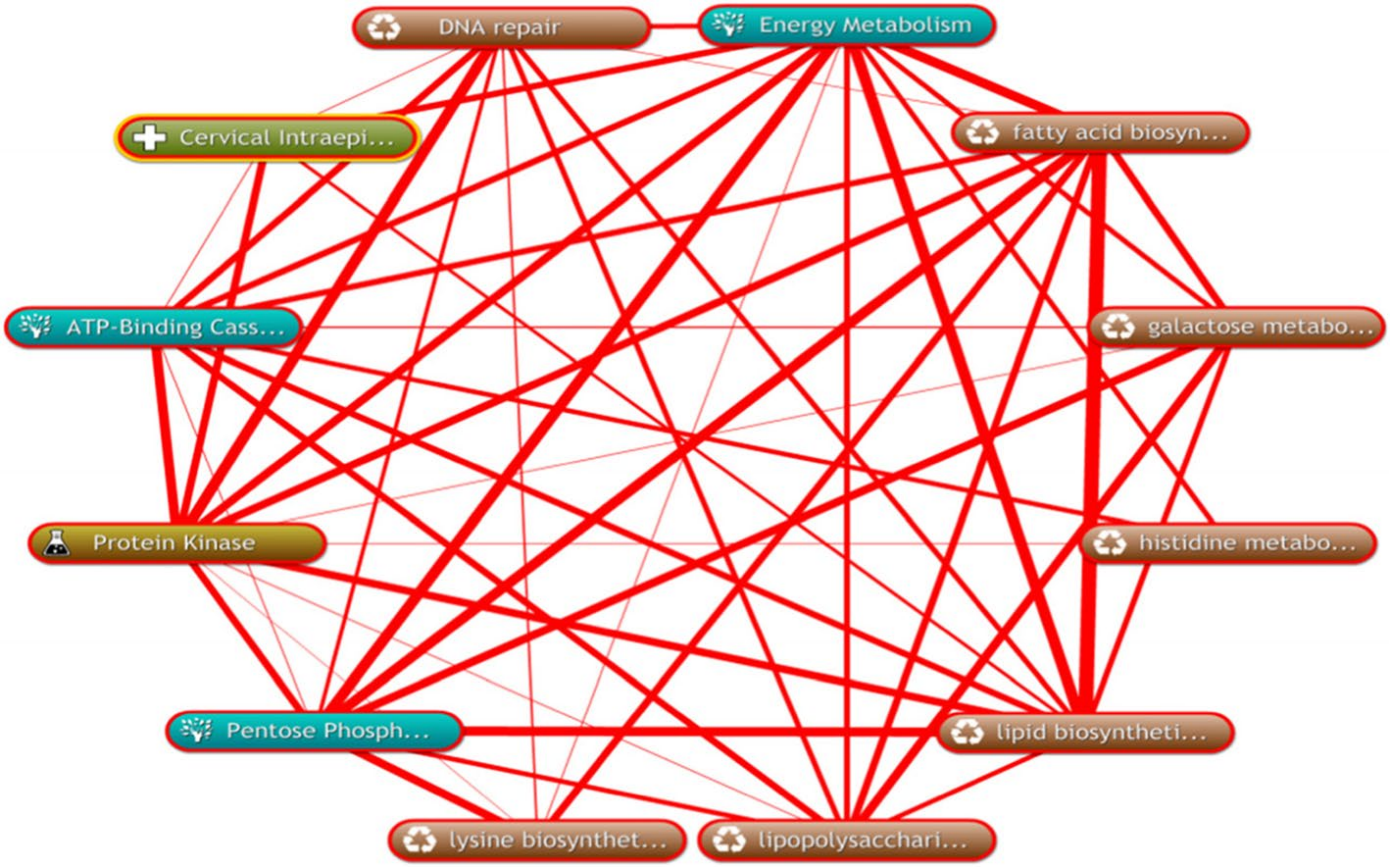
Linear relationship diagram between differential pathway and CIN

**Fig. 4:**
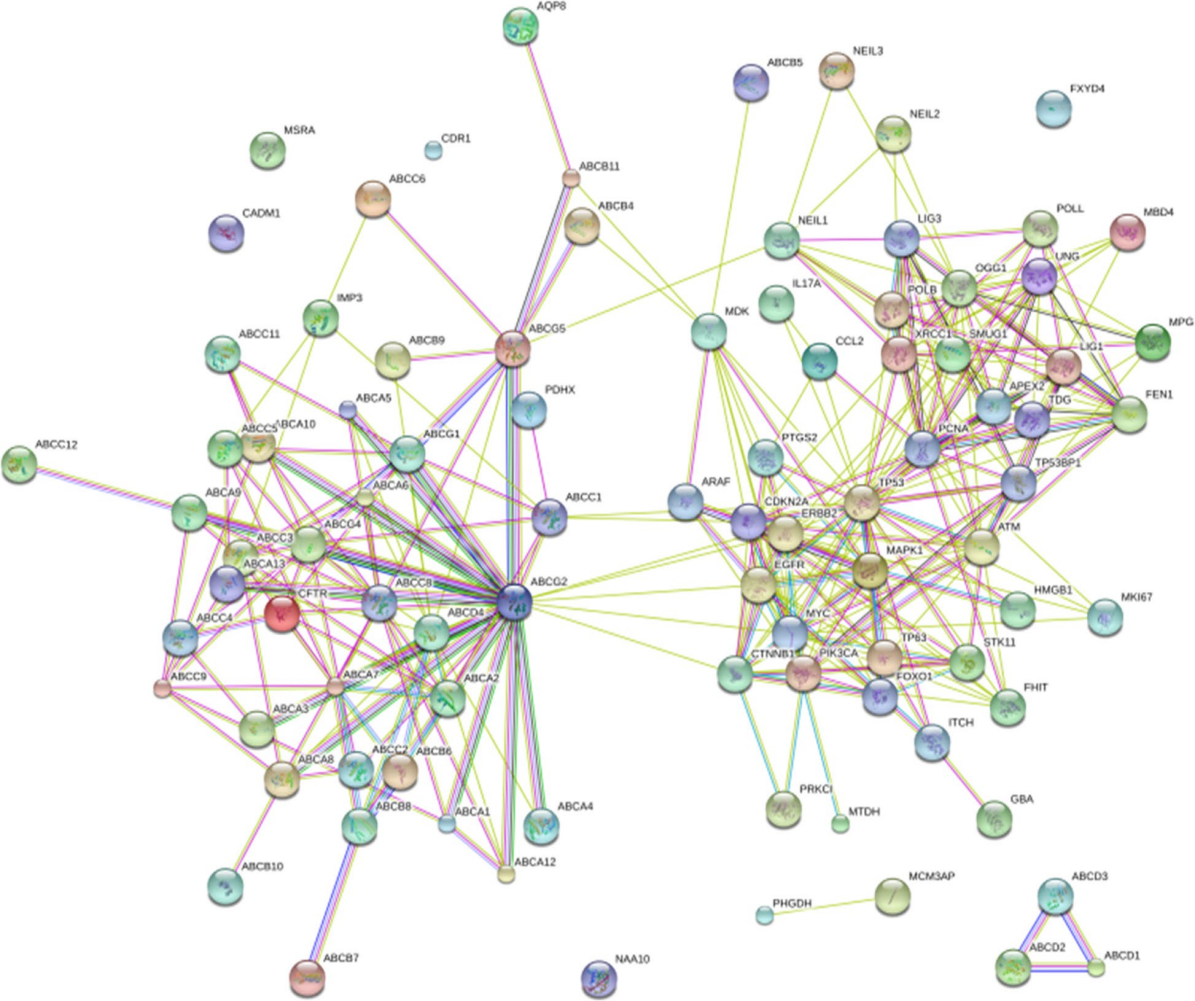
Interaction analysis between gens obtained by differential signaling pathways and CIN progression-related proteein genes

The OTU is classified into 23 classes, 41 orders, 56 families, and 73 genera in several classification levels of class, order, family, and genus. The species abundance of each sample is shown in Fig. 2 and Fig. 3. Using PICRUSt (Phylogenetic Investigation of Communities by Reconstruction of Unobserved States) software for species abundance analysis, combined with KEGG data, the results show that: the genes contained in the CIN group are mainly involved in the following pathways: Transporters, ABC transporters, DNA repair and recombination proteins, General function prediction only, Purine metabolism, Two-component system (Fig. 4).

### COREMINE biological annotation of differential pathways

The biological annotations of the screened differential pathways were carried out using COREMINE to further explain the relationship between the differential pathways and CIN progression. As shown in the figure, using the differential pathway name and CIN (cervical intraepithelial neoplasia) as keywords to conduct a co-occurrence analysis of the literature, a total of 5 differential pathways were found in the annotation in the network: ABC transporters, Base excision repair, Energy metabolism, Lipid biosynthesis proteins, Protein kinases. It is suggested that these differential pathways are related to CIN (Fig. 3).

### Interaction analysis between genes obtained by differential signalling pathways and CIN progression-related protein genes

A total of 79 human genes in the above 5 differential signalling pathways were queried, such as APEX2, POLB, and POLL, among others. Using the String tool (http://string-db.org/), a comprehensive analysis of the protein interaction between 79 genes and 24 known genes related to CIN progression was performed to explain the overall relationship between this gene and CIN progression. The genes that interacted with 24 known genes to form a protein interaction network were ABCC1, ABCC3, ABCC5, ABCC6, ABCG2, APEX2, ARAF, ATM, FEN1, GBA, HMGB1, LIG1, LIG3, MCM3AP, MDK, OGG1, PCNA, POLB, SMUG1, TDG, UNG, and XRCC1 (Fig. 4), indicating that these genes might be functionally related and suggesting that the selected genes might also be related to CIN. In particular, ATM and 11 genes related to CIN progression (TP53, TP63, EGFR, ERBB2, PIK3CA, CDKN2A, CTNNB1, FHIT, MYC, STK11, TP53BP1) interacted directly; PCNA and 10 genes related to CIN progression Genes (CDKN2A, TP53, EGFR, MYC, CCL2, ERBB2, MKI67, CTNNB1, FHIT, TP53BP1) interacted directly; ABCG2 and 5 genes related to CIN progression (CTNNB1, EGFR, ERBB2, MYC, TP53) interacted directly; MDK and 5 genes related to CIN progression (MAPK1, TP53, ERBB2, PIK3CA, EGFR, MYC, PTGS2) interacted directly.

### Protein-protein interaction network of 5 differential pathway genes

**Fig. 5:**
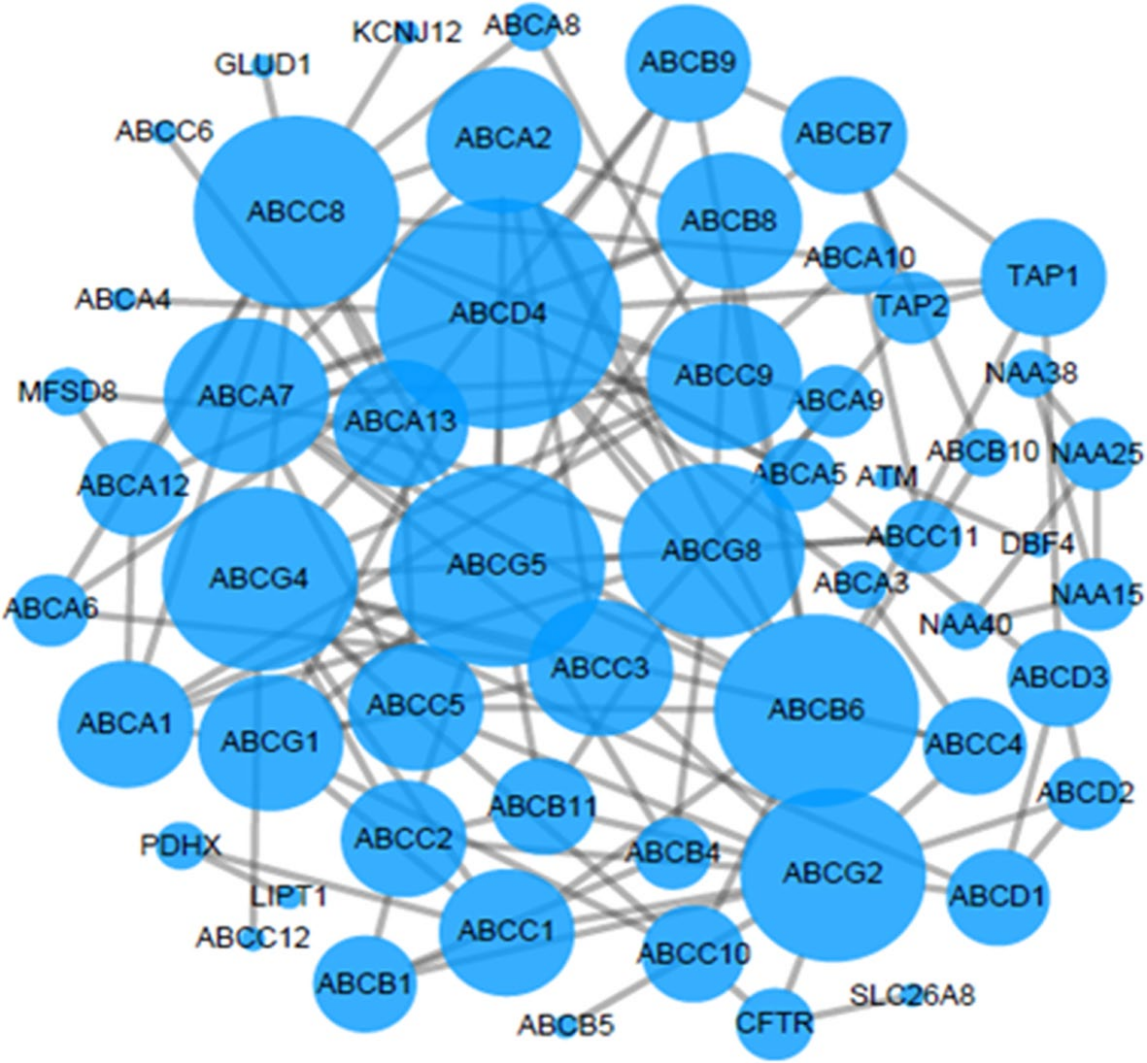
Interaction diagram of ABC transporters pathway genes

**Fig. 6:**
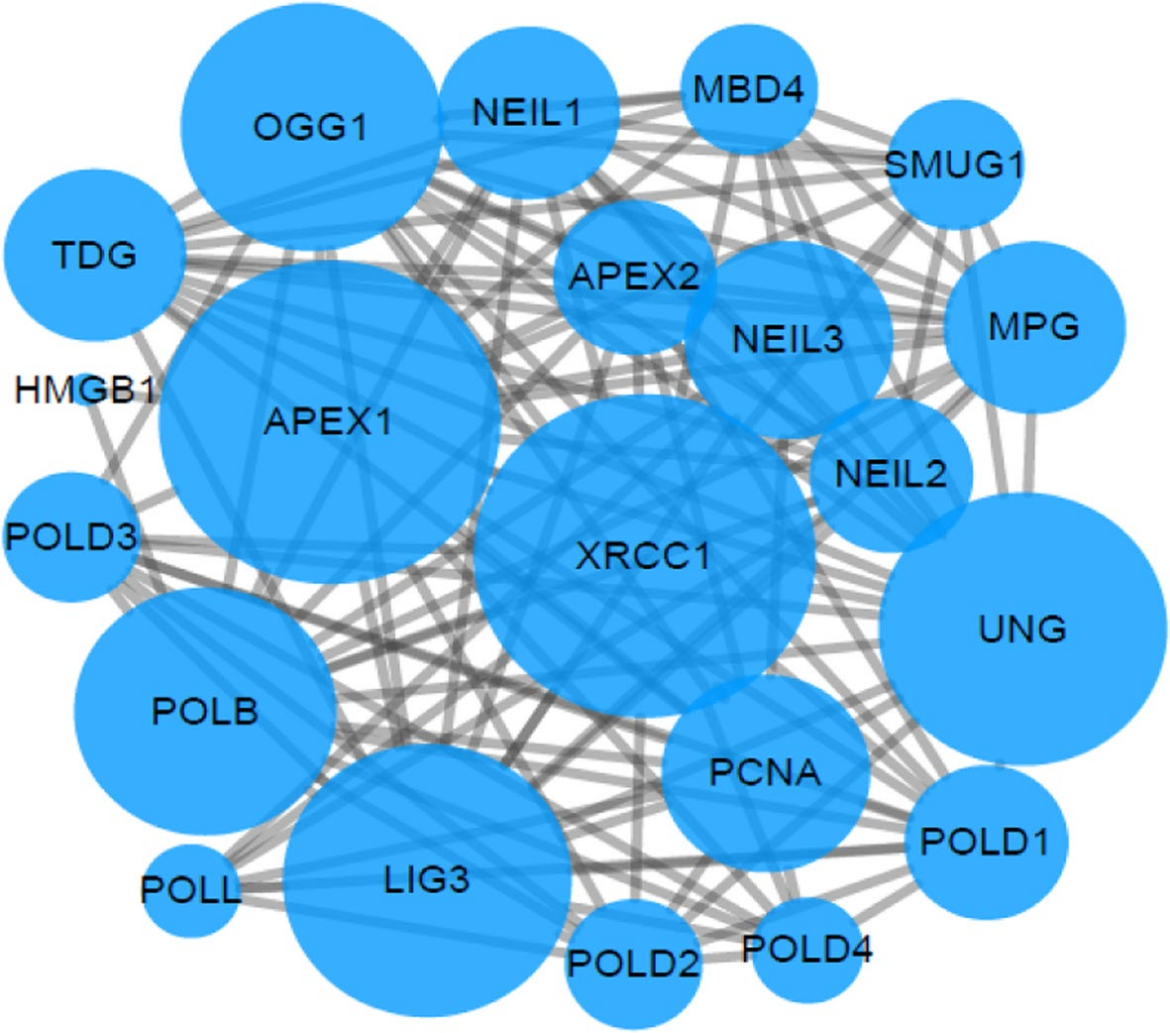
Interaction diagram of base excision repair pathway genes

**Fig. 7:**
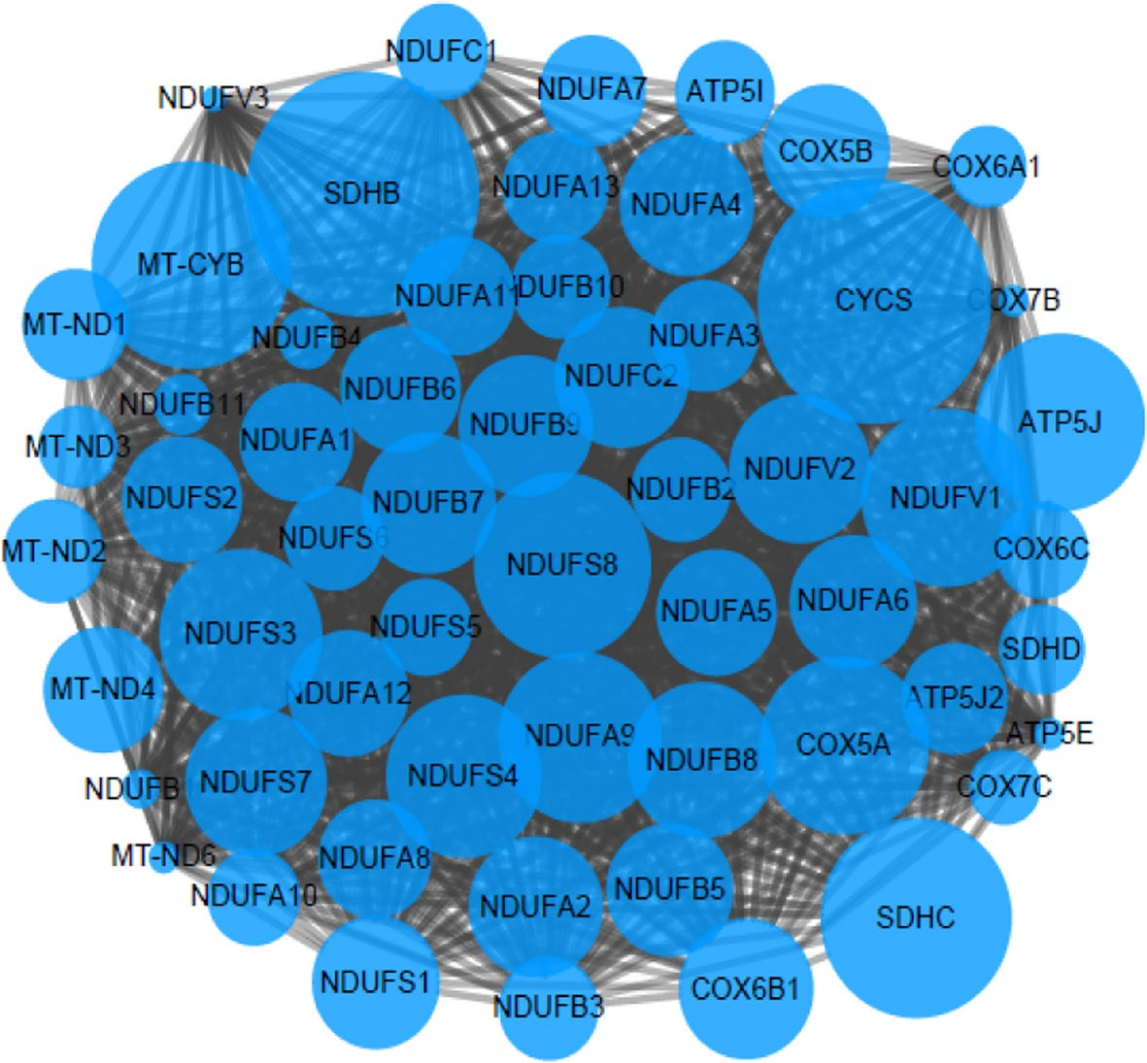
Interaction relationship diagram of energy metabolism pathway

**Fig. 8:**
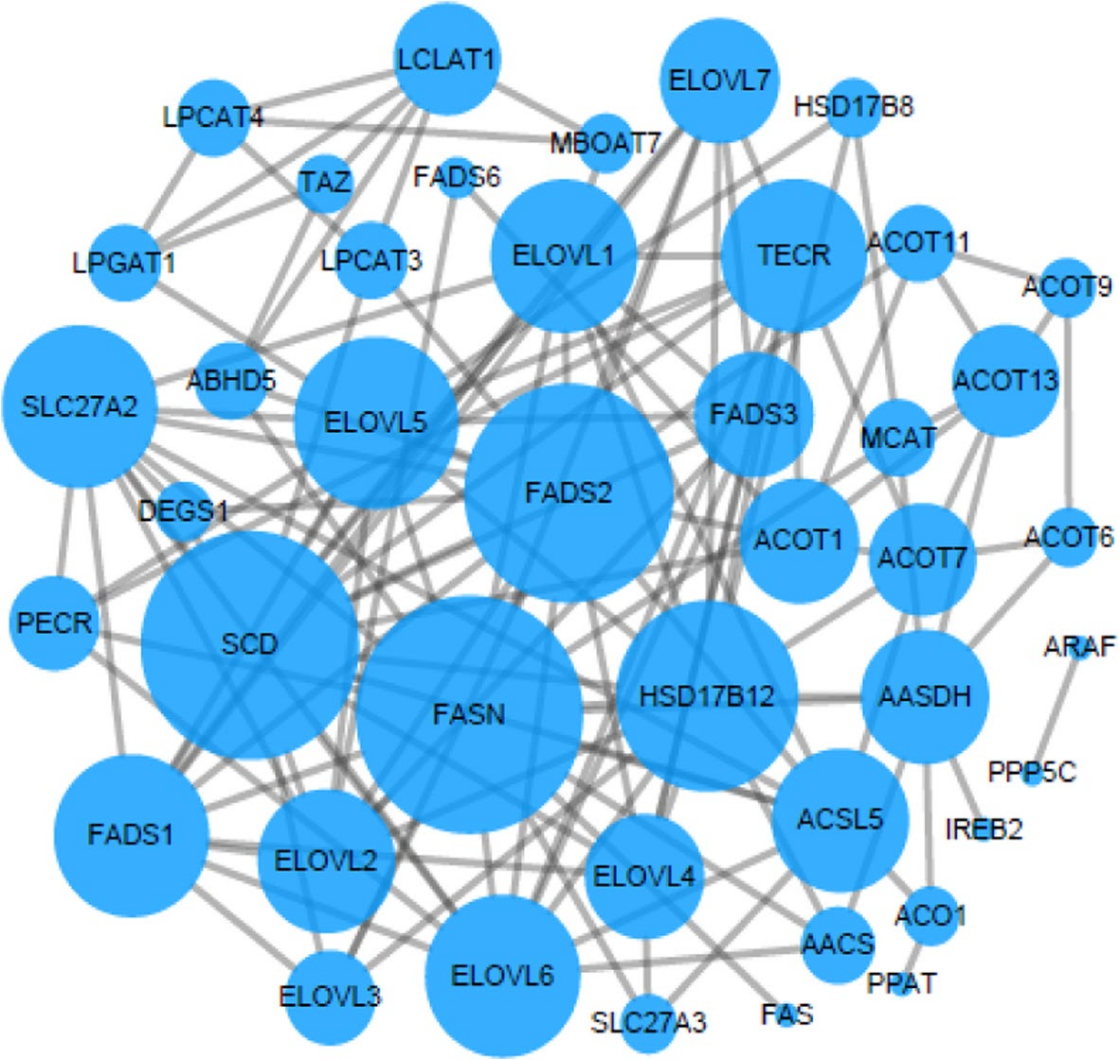
Interaction diagram of lipid biosynthesis protein pathway genes

**Fig. 9:**
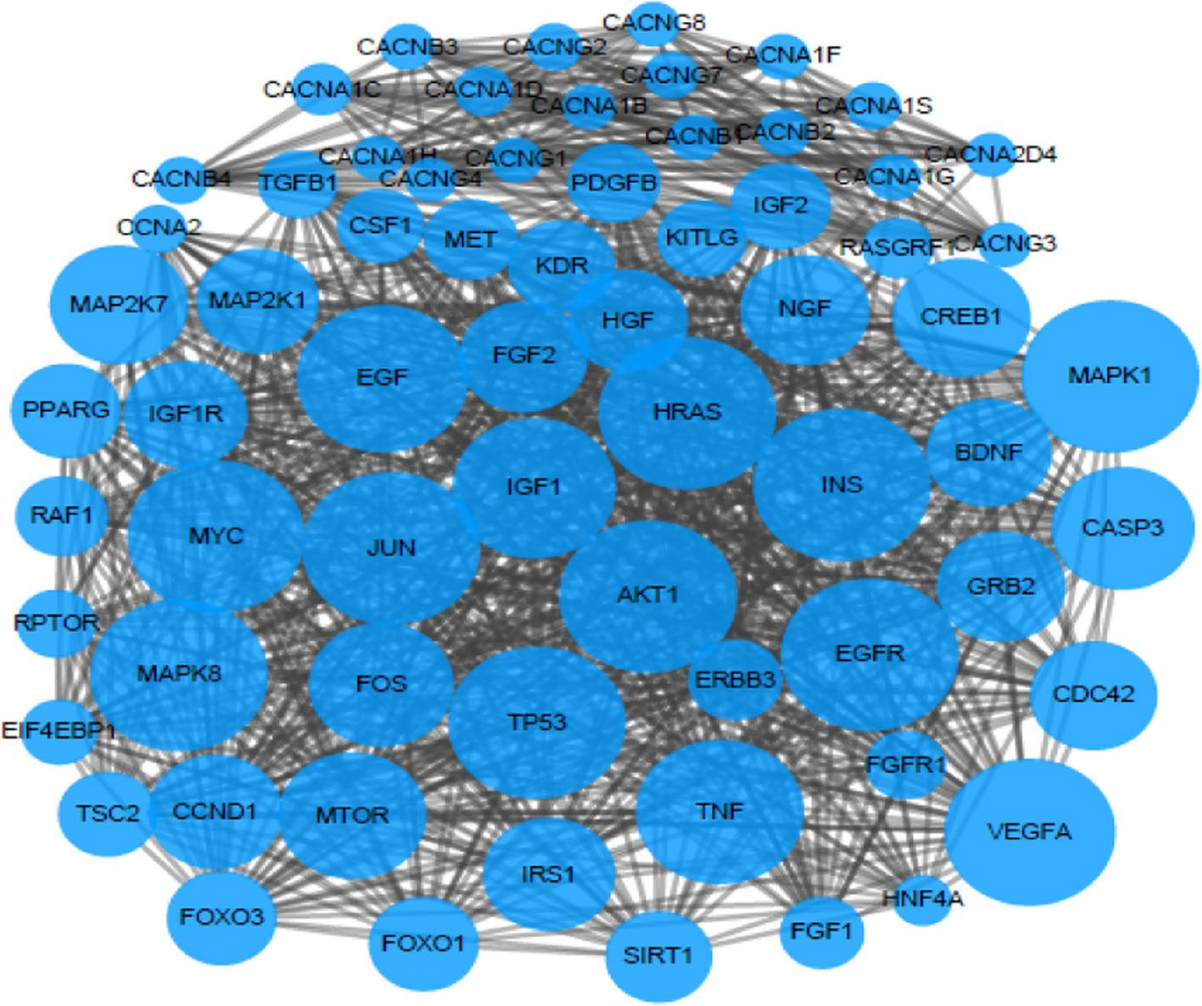
Interaction diagram of protein kinases pathway genes

The STRING online database was used to construct a PPI network diagram for the genes of each pathway, and Cytoscape and MOCDE were applied to visually analyse and cluster the PPI and then obtain 5 protein interaction maps. Among them, the larger the node, the higher was the credibility that the gene was related to other genes in the network. The details are shown in Figs. 5, 6, 7, 8, and 9.

### Literature search and genetic screening results

Through PUBMED, a literature search was conducted with keywords such as gene name, cervical cancer, cervical intraepithelial neoplasia, CIN, cancer, etc., to gain a preliminary understanding of the relationship between genes and tumours. Through protein interaction mapping and a literature search, we initially selected ATM, ABCG2, PCNA, XRCC1, HMGB1, OGG1, LIG1, SMUG1, FEN1, and TDG as candidate differential genes. Among them, ATM and ABCG2 were present in the ABC transporters pathway, and PCNA, XRCC1, HMGB1, OGG1, LIG1, SMUG1, FEN1, and TDG were present in the Base excision repair pathway.

### The relationship between candidate genes and the progress of bacteria and CIN

**Fig. 10:**
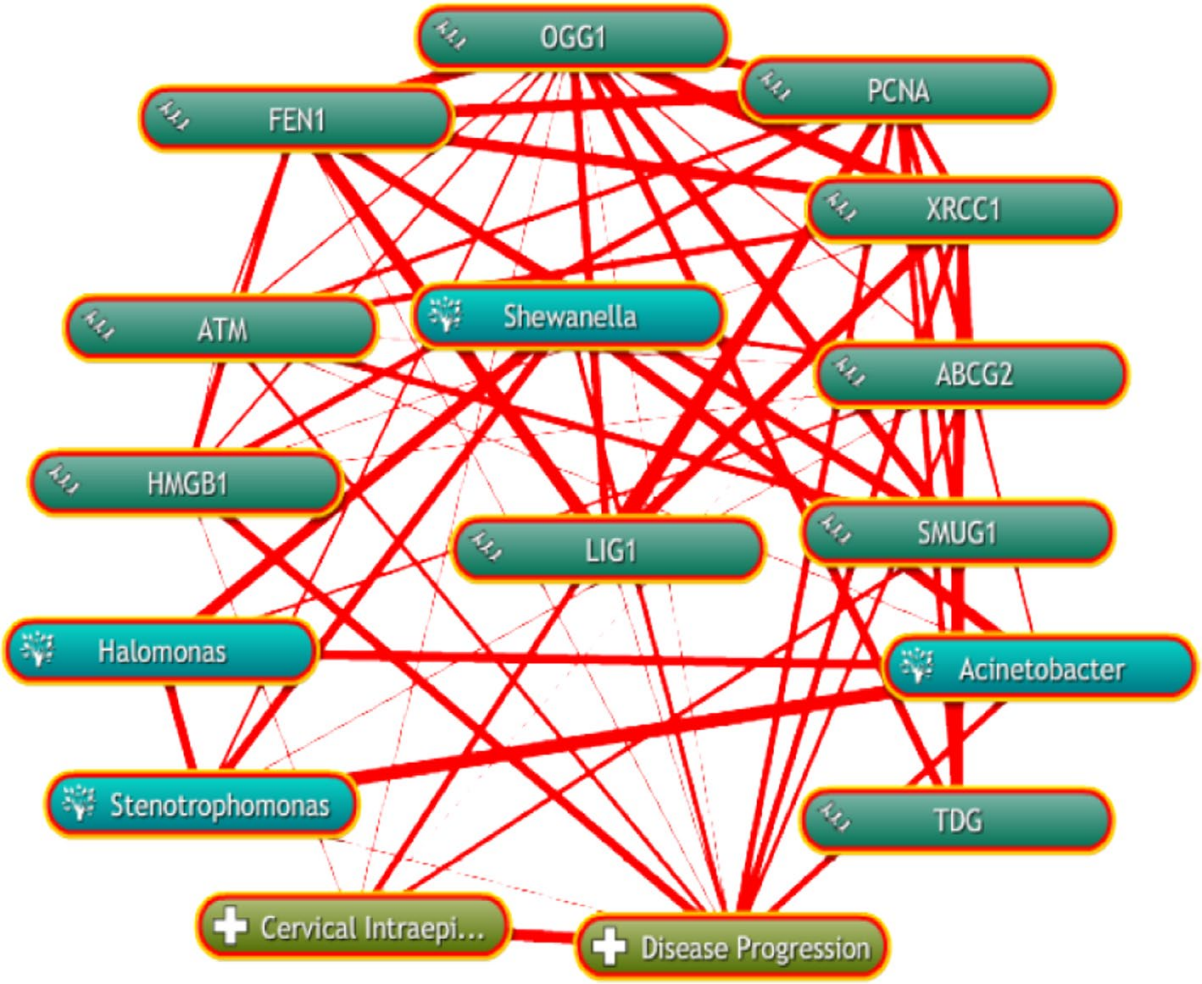
The linear relationship between candidate differential genes and the progress of bacteria and CIN

Biological annotation and text mining were carried out through the COREMINE database to further illustrate the relationship between the 10 candidate differential genes and the progress of bacteria and CIN. As shown in Fig. 10, the co-occurrence analysis of the literature with the key words of gene name, CIN (Cervical intraepithelial neoplasia), bacterial name (Stenotrophom, Chitinophaga, Acinetobacter, Halomonas, Shewanella) and disease progression (Disease Progression) resulted in 10 genes all in the annotation network. Among them, PCNA, HMGB1, OGG1, SMUG1, and XRCC1 had a direct network relationship with the progress of CIN, while ATM, ABCG2, TDG, LIG1, and FEN1 had an indirect relationship. Excluding Chitinophaga, information on which could not be found, the remaining four bacterial genera were researched. The figure shows a complex network relationship between candidate genes and the progress of bacteria and CIN.

### Expression verification of candidate genes

#### Patient clinical characteristics

Among the 38 normal, 52 CIN and 30 cervical cancer patients, average age was 43.55 ± 9.54, 42.12 ± 9.67, and 49.10 ± 10.49 years. The average age of the cervical cancer group was higher than the normal and CIN groups (P<0.05), and the number menopausal patients was greater in the cervical cancer group than the normal and CIN groups (P<0.05). The HPV infection rate in the normal group, CIN group, and cervical cancer group was 39.47, 90.38, and 93.33%, respectively. The HPV infection rate gradually increased with the degree of cervical lesions (*P* < 0.05). TCT also increased with the severity of cervical lesions (P < 0.05). However, there were no significant differences in the pregnancy and parity numbers among the included cases (*P* > 0.05). Age, menopause, HPV infection, and the severity of TCT might affect the occurrence and development of CIN (Table 5).

**Table 5:** Clinical characteristics of patients in each group

	Normal (*n* = 38)	CIN (*n* = 52)	Cervical cancer (*n* = 30)	F/X^2^	*P* value
Age	43.55 ± 9.54	42.12 ± 9.67	49.10 ± 10.49	4.959	**0.009**
Pregnancy	3.11 ± 1.98	3.44 ± 1.85	4.00 ± 1.44	2.079	0.130
parity	1.66 ± 0.85	1.67 ± 1.34	2.20 ± 1.24	2.302	0.105
Menopause				13.752	**0.001**
Yes	9	10	17		
No	29	42	13		
HPV				37.521	**0.000**
Positive	15	47	28		
Negative	23	5	2		
TCT				99.678	**0.000**
Normal	23	1	0		
ASCUS	6	14	4		
LSIL/HSIL	8	36	10		
SCC	1	1	16		

#### Comparison of target gene mRNA expression in different cervical tissues

**Fig. 11:**
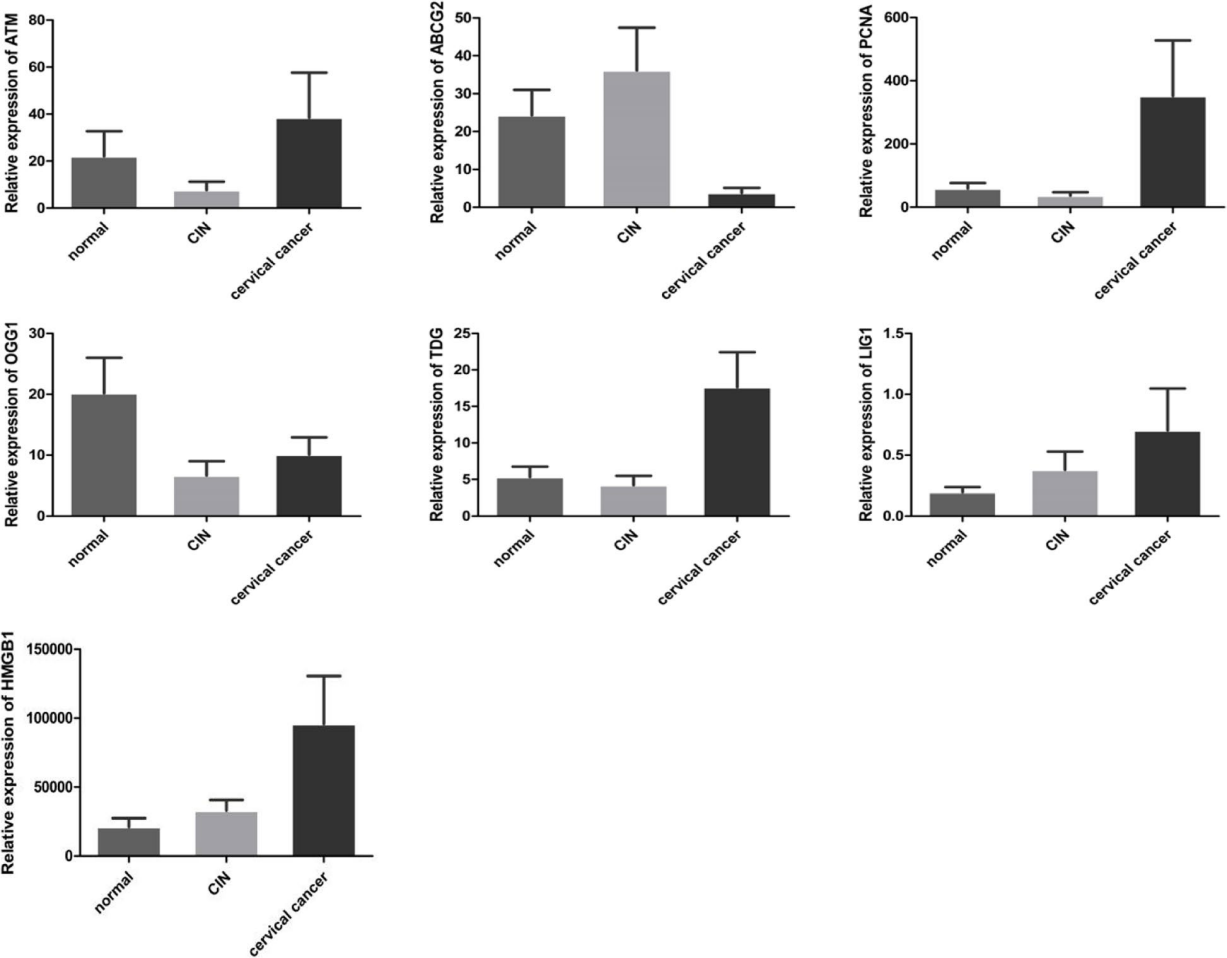
ATM, ABCG2, PCNA, OGG1, TDG, LIG1, and HMGB1 relative expression in different cervical tissues

The fluorescence quantitative PCR results showed that ATM, ABCG2, PCNA, OGG1, TDG, LIG1, and HMGB1 expression levels were statistically significant in three different cervical tissues (*P* < 0.05); There were no differences in the expression levels of XRCC1, SMUG1, and FEN1 (*P* > 0.05). Among them, The expression of ATM was higher in the cervical cancer group than the CIN group (*P* < 0.05). There was no difference between the normal and CIN group and the cervical cancer group (*P* > 0.05). The expression levels of PCNA, TDG, and LIG1 were elevated in cervical cancer group compared with the normal and CIN groups (*P* < 0.05). There were no differences between the normal group and CIN group (*P* > 0.05). The expression of ABCG2 was lower in the cervical cancer group than the normal and CIN groups (*P* < 0.05). There were no differences between the normal and CIN groups (*P* > 0.05). The expression level of OGG1 was lower in the CIN group than the normal group (*P* < 0.05) but higher than in cervical cancer group (*P* < 0.05). Compared with the cervical cancer group, the normal group showed no differences (*P* > 0.05). The expression of HMGB1 gradually increased with the aggravation of cervical lesions (*P* < 0.05). See Table 6 and Fig. 11 for details.

**Table 6:** Real-time fluorescence quantitative PCR detection of target gene mRNA expression in each tissue (copies/μl RNA x ± S)

Gene Name	Normal group (*n* = 38)	CIN group (*n* = 52)	Cervical Cancer Group (*n* = 30)	*P* value
ATM	22.07 ± 65.65	7.70 ± 25.82	38.48 ± 104.85	*P* = 0.074* *P* = 0.294** ***P*** = 0.005***
ABCG2	24.33 ± 41.26	36.08 ± 81.64	3.91 ± 7.22	*P* = 0.133* ***P*** = 0.018** ***P*** = 0.000***
PCNA	59.18 ± 108.64	37.00 ± 74.94	351.83 ± 960.67	*P* = 0.967* ***P*** = 0.033** ***P*** = 0.004***
OGG1	20.18 ± 35.81	6.64 ± 16.88	10.08 ± 15.49	***P*** = 0.003* *P* = 0.786** ***P*** = 0.008***
TDG	5.18 ± 8.83	4.22 ± 9.09	17.63 ± 26.20	*P* = 0.053* ***P*** = 0.031** P = 0.000***
LIG1	0.20 ± 0.26	0.38 ± 1.08	0.70 ± 1.88	*P* = 0.546* ***P*** = 0.010** ***P*** = 0.001***
HMGB1	21,235.07 ± 38,403.32	33,052.50 ± 54,685.92	95,839.75 ± 190,013.59	***P*** = 0.039* P = 0.001** ***P*** = 0.038***
XRCC1	1.08 ± 1.97	2.27 ± 5.24	1.76 ± 4.74	*P* = 0.270* *P* = 0.776** *P* = 0.476***
SMUG1	57.19 ± 238.93	85.84 ± 189.57	100.67 ± 301.45	*P* = 0.452* *P* = 0.466** *P* = 0.900***
FEN1	30.05 ± 41.93	24.29 ± 43.85	79.65 ± 193.15	*P* = 0.508* *P* = 0.970** *P* = 0.637***

* represents the comparison between the normal group and the CIN group, ** represents the comparison between the normal group and the cervical cancer group, and *** represents the comparison between the CIN group and the cervical cancer group

#### ROC curve analysis of differential genes with respect to the occurrence and progression of early warning CIN

ROC curve analysis showed the highest value for genetic OGG1 for early warning signs of CIN, with an AUC of 0.68 (*P* < 0.05). The HMGB1 gene had an AUC of 0.63 (*P* < 0.05). The sensitivity of OGG1 and HMGB1 as warning signs of CIN was 57.7 and 75.0%, and the specificity was 78.9 and 57.9%, respectively. The values for the residual genes TDG, ATM, ABCG2, LIG1, and PCNA revealed no association with CIN. Early warning of the progress of CIN was observed for HMGB1, LIG1, ABCG2, TDG, and PCNA with an AUC of 0.74, 0.68, 0.67, 0.65, and 0.65 (*P* < 0.05). The sensitivity was 86.7, 83.3, 96.7, 50.0, and 76.7%, and the specificity was 57.9, 52.6,39.5, 81.6 and 57.9%, respectively. The values for the residual genes ATMand OGG1 showed no association with early warning of CIN progress. Genes with value for the occurrence and progression of CIN early warning signs were OGG1 and HMGB1 and HMGB1, LIG1, ABCG2, TDG, and PCNA, respectively. HMGB1genes were valuable for early warning of the occurrence and progression of CIN (see Tables **7** and **8** for details).

**Table 7:** The AUC value, sensitivity, and specificity of differentially expressed genes for early warning of CIN occurrence

Genetics	Sensitivity(%)	Specificity (%)	AUC (95% CI)	*P* value
ATM	63.5	60.5	0.61 (0.50–0.71)	0.069
ABCG2	88.5	36.8	0.59 (0.49–0.70)	0.142
PCNA	80.8	36.8	0.50 (0.40–0.61)	0.969
OGG1	57.7	78.9	0.68 (0.58–0.78)	**0.001**
TDG	78.8	55.3	0.62 (0.51–0.72)	0.054
LIG1	69.2	47.4	0.54 (0.43–0.64)	0.547
HMGB1	75.0	57.9	0.63 (0.52–0.73)	**0.037**

**Table 8:** The AUC value, sensitivity and specificity of differentially expressed genes for early warning of CIN progression

Genetics	Sensitivity(%)	Specificity (%)	AUC (95% CI)	*P* value
ATM	36.7	81.6	0.58 (0.45–0.69)	0.296
ABCG2	96.7	39.5	0.67 (0.55–0.78)	**0.010**
PCNA	76.7	57.9	0.65 (0.53–0.76)	**0.029**
OGG1	33.3	52.6	0.52 (0.40–0.64)	0.787
TDG	50.0	81.6	0.65 (0.53–0.77)	**0.027**
LIG1	83.3	52.6	0.68 (0.56–0.79)	**0.005**
HMGB1	86.7	57.9	0.74 (0.62–0.84)	**0.0001**

#### Logistic regression analysis of risk factors for CIN occurrence and progression

##### Logistic regression analysis that promotes risk factors for CIN

Age, menopause, HPV infection, TCT and ROC analysis of meaningful genes were subjected to single-factor logistic regression analysis, and meaningful factors were then analysed using multi-factor logistic regression analysis. The results revealed that during the progression of the normal cervix to CIN, HPV positivity and TCT severity were risk factors for CIN (see Table 9 for details).

**Table 9:** Logistic regression analysis of risk factors that promote CIN

	Single-factor logistic regression analysis			Multiple-factor logistic regression analysis		
	OR	95% CI	*P* value	OR	95% CI	*P* value
Age	0.984	0.942–1.029	0.481	**–**	**–**	**–**
Menopause	0.767	0.277–2.122	0.610	**–**	**–**	**–**
**HPV**	14.413	4.664–44.545	**0.000**	16.702	4.308–64.754	**0.000**
**T CT**	7.948	.0473–20.732	**0.000**	9.390	.7752–31.768	**0.000**
OGG1	0.979	0.959–0.999	**0.039**	0.990	0.961–1.020	0.505
H MGB1	1.006	0.995–1.016	0.270	**–**	**–**	**–**

Note: “-” indicates no such item

##### Logistic regression analysis of risk factors for CIN progression

Age, menopause, HPV infection, TCT and ROC analysis of meaningful genes were subjected to single-factor logistic regression analysis, and meaningful factors were then subjected to multi-factor logistic regression analysis. The results showed that the low expression of ABCG2 and high expression of PCNA and TDG were risk factors for the progression of CIN (see Table 10 for details).

**Table 10:** Logistic regression analysis of risk factors for CIN progression

	Single-factor logistic regression analysis			Multiple-factor logistic regression analysis		
	OR	95% CI	*P* value	OR	95% CI	*P* value
Age	1.070	1.020–1.122	**0.005**	0.992	0.917–1.073	0.849
Menopause	4.800	1.775–12.983	**0.002**	3.067	0.529–17.784	0.211
HPV	1.489	.2710–8.196	0.647	**–**	**–**	**–**
TCT	1.333	.4470–3.977	0.606	**–**	**–**	**–**
**ABCG2**	0.903	0.833–0.978	**0.012**	0.869	0.791–0.956	**0.004**
**PCNA**	1.005	1.000–1.010	**0.048**	1.011	0.998–1.024	**0.036**
HMGB1	1.005	0.999–1.011	0.074	**–**	**–**	**–**
LIG1	1.171	.8400–1.633	0.352	**–**	**–**	**–**
**TDG**	1.057	1.013–1.103	**0.011**	1.316	1.019–1.700	**0.035**
Note: “-” indicates no such ite						

#### Establishment andselection of Random Forest Models

According to the results of the multivariate logistic regression analysis, the risk factors promoting the occurrence and development of CIN, such as HPV positivity, TCT severity, ABCG2, PCNA and TDG genes, were used to establish random forest models with different combinations of these indicators. A total of 7 models were established, as shown in Table 11. According to the accuracy, AUC value and OOB error value of each model, the best model was selected. Among the various models, the model 4 accuracy rate and AUC value were relatively large, and the OOB error value was the smallest. Therefore, model 4 (ABCG2 + PCNA+TDG) was selected as the best early warning model for the occurrence and progress of CIN.

**Table 11:** The occurrence and progression of CIN evaluated by the SCC random forest model

Number	Inclusion of indicators	Specific projects	Accuracy	AUC	OOB error
1	All clinical factors	age + menopause+HPV + gravidity+parity+TCT	56.41	51.78	28.95%
2	Differential gene	ATM + ABCG2 + PCNA+OGG1 + TDG + LIG1 + HMGB1	84.61	84.02	18.42%
3	Differential gene + clinical factors	ATM + ABCG2 + PCNA+OGG1 + TDG + LIG1 + HMGB1 + age + menopause+HPV + TCT	82.05	87.28	23.68%
4	**Single-factor logistic regression P < 0.05 gene**	**ABCG2 + PCNA + TDG**	**84.62**	**83.14**	**18.4%**
5	The difference in single factor logistics + 0.10 gene	ABCG2 + PCNA+TDG + age + menopause+HPV + TCT	76.92	83.73	23.68%
6	Multiple-factor logistic regression P < 0.05 gene	ABCG2 + TDG	79.49	85.50	21.05%
7	Multiple-factor logistic regression P < 0.05 gene + different clinical factors	ABCG2 + TDG + age + menopause+HPV + TCT	84.62	82.54	23.68%

## Discussion

### The relationship between microbiology and CIN progression

Persistent high-risk human papillomavirus infection is undoubtedly the main carcinogen leading to CIN and cervical cancer. However, studies have found that not all patients with cervical HPV infection experience development into cancer [1]. Although it is now believed that auxiliary factors other than HPV play a key role in the development of tumours, most of the potential mechanisms of this carcinogenic effect are still unknown [2]. These carcinogenic factors include genetic factors, environmental factors, immune function, cellular defense, and body-specific genes and cytokines [9]. Evidence shows that the cause of human diseases is not only a single pathogen, but also involves the overall changes in the human microbiology group [3]. However, few studies have examined the progress of the microbiome in CIN at this stage. The available small numbers of cervical and vaginal microbiological studies based on high-throughput sequencing have mainly focused on other populations. The structural characteristics of the cervical flora in the normal and disease states in the Chinese population are not clear. Therefore, in this study, normal cervical tissues, CIN and cervical cancer patients in southern China served as the research objectives. Using high-throughput sequencing analysis technology based on bacterial 16S rDNA, the composition of the cervical flora was analysed and compared in the above population using bioinformatics methods. To construct an atlas of the composition of the significant specific flora of the disease, we selected specific flora or species that were significantly related to the disease. This project had the following main research goals: 1. preliminary understanding of the structure and diversity of the bacterial community in normal cervical tissue; 2. preliminary understanding of the structure of the bacterial community in patients with CIN and cervical cancer without treatment by pathology and diversity characteristics; 3. Attempt to find and identify functional pathways of genes present in related flora or species related to CIN progression.

### The difference and clinical significance of the main bacterial groups among CIN, cervical cancer and normal cervix

In this study, Illumina’sMesiq high-throughput sequencing platform was used to conduct high-throughput analysis of cervical tissue-related bacterial communities, and the data obtained were subjected to quality control such as removal of single reads; qualified sequences were annotated by OTUs and evaluated by inter-sample diversity analysis such as dilution curves, among others. We confirmed that the amount of sequencing in this study was sufficient to cover cervical bacterial species, and the sample richness and uniformity between groups were good. In addition, there were no significant differences in age, parity, or contraceptive method, among others, in the selected cases, and some interfering factors were excluded. In general, cervical tissues were identified from 23 bacterial strains and 73 genera. Regarding the level distribution of vaginal-related bacterial communities, studies [10] have indicated that the phyla Firmicutes, Actinobacteria, Bacteroidetes, and Proteobacteria, and non-wall bacteria Tenericutes, Fusobacteria and TM7, are the main microorganisms. It has also been reported that HSIL vaginal microorganisms are characterized by higher levels of Sneathiasanguinegens, Anaerococcustetradius and Peptostreptococcusanaerobius, and lower levels of LSIL in Lactobacillus jannaschii. We found that the main microorganisms in CIN1/2 were Stenotrophom, Chitinophaga, and Acinetobacter, and the main microorganisms in CIN3 and cervical cancer were Halomonas, Shewanella, Acinetobacter. By comparison with the results of several existing high-throughput sequencing analyses of vaginal flora [4, 10, 11], the relative abundance of cervical bacterial communities at the phylum and genus levels and other species revealed certain differences, considering the possible relationships with factors such as disease state, race, and region.

Studies have shown that Lactobacillus is the most dominant bacteria in the vagina [12]. Symbiotic vaginal Lactobacillus is believed to produce species-specific metabolites and bacteriocins by maintaining a constant pH value, thereby destroying pathogen biofilms [13]. However, most current studies on the microbiota focus on the vaginal microbiota, and there are few reports on the microbiome in cervical tissue. At the subordinate level of this study, Lactobacillus was still dominant in most of the normal group and CIN group, but the proportion was significantly reduced in the CIN and cervical cancer groups. This result further supports the ability of Lactobacillus to maintain a normal vagina and cervix and provide protection from the environment. However, a small number of samples in the normal group still lacked a dominant Lactobacillus genus, potentially due to the differences between cervical tissue and vaginal microbes. The small sample size was not ruled out, which will be expanded in future detailed research.

The role of the microbiome in the progression of CIN is gradually becoming recognized. MitraA [4] recently performed 16S rRNA gene amplification of the vaginal wall microbes of 52 cases of LSIL, 92 cases of HSIL, 5 cases of ICC and 20 cases of normal controls. The results showed that the vaginal microbial diversity was associated with the severity of CIN disease. We can participate in regulating the persistence of viral infections and disease progression. However, no reports have examined the mechanisms and gene functions involved in the progression of CIN related to the cervical microbiome. The 16S rRNA is an important tool for microbial community research, but it does not provide direct evidence of community function. In 2013, Langille et al. [8] described a software that uses marker gene data and reference genome databases to predict the functional composition of metagenomics, namely PICRUSt (Phylogenetic Investigation of Communities by Reconstruction of Unobserved States). PICRUSt uses the extended ancestral state reconstruction algorithm to predict the presence of specific gene families, and then combines the gene families to estimate the compound metagenomics. Using 16S information, PICRUSt can retrieve key results from the human microbiome and accurately predict the abundance of gene families associated with the host and environmental communities. In 2016, Tseng and other scholars [14] used 16S rRNA sequencing to identify the microbiome changes in gastric cancer tissues before and after surgery, and PICRUSt software to predict the classification of gene functional groups related to the microbiome. In this study, PICRUSt software was used to analyse the selected pathways, and the obtained differential pathway information was biologically annotated by COREMINE. Five differential pathways were found: ABC transporters, Base excision repair, Energy metabolism, Lipid biosynthesis proteins, Protein kinases Exist, suggesting that these 5 differential pathways were related to CIN. Using the String tool, among these differential signalling pathways, the human genes ATM and PCNA showed the closest relationship with the progression of CIN. We will further examine the relationship between these pathways and related genes and the progression of CIN to inhibit the progression of this disease.

Although HPV infection is common in sexually active women, most infections are temporary. Only some women infected with the HPV wasprogressed to precancerous lesions and invasive cervical cancer. A large number of research reports suggest that persistent high-risk human papillomavirus (hrHPV) infection is the greatest risk factor for the occurrence and development of CIN and cervical cancer. The mechanism by which HPV infection continues to cause cancer is not well understood. Studies have suggested that the hrHPV subtype itself produces two proto-oncoproteins, E6 and E7, which are key to its pathogenesis [15]. E6 and E7 proto-oncoproteins can change the terminal differentiation of host keratinocytes and combine with host cell cycle regulatory proteins to destroy the negative regulation of the cell cycle, leading to abnormal cell cycle regulation [16]. Additionally, normal tissues eventually develop into CIN and cervical cancer. Recently, Hu Z et al. [17] used whole-genome sequencing and capture sequencing to determine for the first time the impact of HPV-infected human specific integration sites and key fragile integration sites on tumour cells. Whole-genome analysis of HPV integration in CIN and cervical cancer is expected clarify the carcinogenic mechanism and block the occurrence and development of CIN.

### The relationship between candidate genes screened by differential pathways and CIN and cervical cancer

In this study, five differential pathways related to the progression of CIN in the cervical microbial community were analysed. The KEGG database was used to search for the pathway genes, and approximately 1442 genes were queried. The STRING online database was used to construct a PPI network for the genes in each pathway. Cytoscape was used to visualize the network diagram, and then MOCDE was applied to further screen the genes. Combined with the literature search, 10 candidate differential genesrelated to CIN and cervical cancerwere initially screened: ATM, ABCG2, PCNA, XRCC1 HMGB1, OGG1, LIG1, SMUG1, FEN1, and TDG.

A literature search revealed that ATM is highly expressed in cervical cancer, and ATM expression inhibition can enhance the sensitivity of cervical cancer to radiotherapy and chemotherapy [18]. In addition to being related to cervical cancer, ATM is also related to other malignant tumours. For example, Santarpia et al. [19] found that low ATM expression in breast cancer was related to a high DNA mutation rate, tumour progression and increased angiogenesis, and ATM expression was related to patient prognosis. Kim et al. [20] usedimmunohistochemical analysis of 321 gastric cancer patients and found low ATM levels in 205 gastric cancer patients and high expression in 116 gastric cancer patients. The 5-year survival rate and overall survival rate of patients in the ATM low expression group were compared. The patients in the ATM high expression group showed worse outcomes (*P* = 0.017, *P* = 0.027).

ABCG2 is a member of the G subfamily of the ATP-binding cassette (ABC) transporter superfamily. It is a multidrug resistance gene, also known as breast cancer resistance protein (BCRP), and it is found in a variety of malignant tumours. High expression was found in, e.g., liver cancer and pancreatic cancer [21, 22], but low expression in colorectal and cervical cancer [23].

PCNA is the core protein in DNA synthesis, replication, and damage repair and a key factor in cell cycle regulation. It can be used as an indicator of tumour cell proliferation and tumour prognosis. Studies have shown that the expression of PCNA in cervical tissues increases with the increase in CIN and cervical cancer grade [24]. A systematic review showed that the expression of PCNA is closely related to the staging and prognosis of cervical cancer. Cervical cancer with high PCNA expression has a lower survival rate and a worse prognosis [25]. In addition to its high expression in cervical cancer, PCNA is highly expressed in rapidly dividing tumour cells, which in most cases is related to poor prognosis and is an effective target for tumour treatment [26].

HMGB1 is a member of the HMG superfamily. By participating in the functions of cell DNA recombination, replication, repair, and gene transcription, it is widely involved in tumorigenesis, growth, invasion and metastasis. The literature also indicates that HMGB1 is highly expressed in cervical cancer tissues [27]. In ovarian and gastric cancer, HMGB1 knockdown can inhibit cancer cell proliferation, migration and invasion [28, 29]. Wu et al. [30] conducted a meta-analysis of the relationship between HMGB1 and various tumours and found that overexpression of HMGB1 was associated with a poor prognosis of various tumours. HMGB1 is a potential marker for the prognosis of various tumours.

OGG1 is an enzyme that specifically recognizes 8-dihydro-8-oxoguanine (8-oxoG) in the body and excises and repairs it. The presence of this enzyme reduces the risk of DNA mutations and tumorigenesis [31]. Kubo et al. [32] detected low OGG1 expression in oesophageal cancer and its association with lymphatic metastasis and tumour staging. This study showed that the DNA repair pathway involved in OGG1 was closely related to oesophageal cancer, but its expression in CIN and cervical cancer has not yet been reported.

At present, most studies have shown that the XRCC1 gene polymorphism is related to the risk of cervical cancer, and few studies have examined its expression in cervical cancer [33]. Abdel-Fatah et al. [34] found that XRCC1 is highly expressed in ovarian cancer and is closely related to tumour staging, platinum resistance, and death outcomes. Patients with positive XRCC1 expression are more likely to have adverse clinicopathological and survival outcomes, which is considered a new predictive marker for ovarian cancer.

FEN1 is a multifunctional nuclease with a special structure. Its gene mutation may cause autoimmune diseases, chronic inflammation, and tumour susceptibility. He et al. [35] found that FEN1 was up-regulated in cervical cancer tissues. The use of FEN1 inhibitors in combination with paclitaxel can significantly improve the efficacy of paclitaxel in cervical cancer. In addition to its high expression in cervical cancer, FEN1 is overexpressed in many tumours such as breast and lung cancer [36, 37].

Few studies have examined LIG1, SMUG1, and TDG in CIN and cervical cancer. At present, their specific expression in cervical cancer has not been described, but they are closely related to other malignant tumours. It is known that abnormal DNA methylation is an important feature of tumorigenesis. Increased demethylation may be involved in tumorigenesis, and TDG plays an important role in DNA demethylation [38]. Yang et al. [39] found low TDG in early breast cancer and its relationship with a poor prognosis of early breast cancer. Zhong et al. [40] used the Whole Transcriptome Association Study (GWAS) to identify a relationship of SMUG1 with the risk of pancreatic cancer, making it a new candidate gene for predicting pancreatic cancer. At present, the literature shows that LIG1 is a DNA ligase that is usually overexpressed in tumours. LIG1 is considered to be necessary for ligating Okazaki fragments during DNA replication. Therefore, it is also necessary for cell survival and the focus of current clinical efforts. Research on DNA ligase inhibitors to inhibit cancer progression have been reported [41].

We used the COREMINE database to further conduct biological annotation and text mining regarding the relationship between these 10 candidate genes and the progress of bacteria and CIN, and we found that the 10 genes were all present in the annotation network. Among them, PCNA, HMGB1, OGG1, SMUG1, and XRCC1 showed a direct network relationship with the progress of CIN, and ATM, ABCG2, TDG, LIG1, FEN1 and CIN progression showed an indirect network relationship. Chitinophaga could not be found, but information on the other four bacterial genera was available. Among them, ABCG2 had a direct network relationship with the other four bacterial genera, ATM, PCNA and Acinetobacter. There was a direct network relationship, and Acinetobacter (Acinetobacter) was related to HPV infection and CIN progression [42]. OGG1 showed a direct network relationship with Stenotrophom. As can be seen from the network diagram in which these genes showed a complex network relationship with the progress of bacteria and CIN. However, this result has not yet clarified the specific mechanism of genes and bacteria and the progress of CIN, nor the specific role of bacteria in the progression of CIN. Maybe these genes are the cancer promoting genes groupin the cervical microbial community. In follow-up studies, we will further verify the expression of the selected candidate differential genes in cervical tissues. We hope to find specific genes and conduct detailed studies to block CIN progression.

### Expression verification and analysis of candidate genes in CIN

As mentioned above, persistent hrHPV infection is the main reason for the occurrence and development of CIN and cervical cancer. Some studies have suggested that the hrHPV subtype itself produces E6 and E7, two key proto-oncoproteins in its pathogenesis. If HPV oncogenes such as E6 and E7 are integrated into the high-risk area of the patient’s chromosome, this process will interfere with tumour suppressor genes such as P53 or PRb, among others, which may cause CIN and even cervical cancer [43]. In our study, analysis of the patients’ clinical data showed that age, menopause, TCT test results, and HPV infection differed among the three groups of patients, and the HPV infection rate gradually increased with the severity of cervical lesions. Additionally, the cervical cancer group was obviously higher than the normal group. The data once again confirmed that the occurrence of cervical lesions was closely related to HPV, consistent with domestic and foreign reports. TCT examination played an important role in early warning of the occurrence of CIN, and TCT examination combined with HPV detection showed important value in the early diagnosis of CIN. Studies have shown that the residual rate, recurrence rate and the incidence of aggressive lesions after CIN conization are related to age and menopause [44], but whether they can promote the occurrence and progression of CIN remains to be examined.

This study verified the expression of 10 candidate differential genes screened from cervical microbes related to CIN progression pathways in different cervical tissues through fluorescence quantitative PCR: ATM, ABCG2, PCNA, OGG1, TDG, LIG1, and HMGB1. The expression in three different cervical tissues was statistically significant (*P* < 0.05). Other reports have also indicated that ATM, PCNA, and HMGB1 are highly expressed in cervical cancer tissues [24, 27, 45], and ABCG2 is expressed at low levels in cervical cancer tissues [23]. The results of this experiment are consistent with those reports. However, expression levels of TDG, LIG1 and OGG1 have not been examined in cervical cancer tissues. In this experiment, we found that TDG and LIG1 were expressed at high levels in cervical cancer, and OGG1 expression was low in cervical cancer (P < 0.05).

Through ROC curve analysis, we found that the genes that were valuable for early warning of CIN occurrence and progress were OGG1 and HMGB1 and HMGB1, LIG1, ABCG2, TDG, and PCNA, and the gene with value for early warning of CIN occurrence and progress was HMGB1. This result is further confirmed that these genes are the key carcinogenic genes in the cervical microbial community. Additionally, the expression of HMGB1 increased with the severity of cervical lesions, suggesting that HMGB1 was closely related to the occurrence and progression of CIN, and HMGB1 might be a new marker for early warning of the occurrence and progression of CIN. We further searched the literature to understand the expression of HMGB1 in various tumours, and we found that HMGB1 was expressed at high levels in other tumours such as ovarian cancer and gastric cancer; overexpression of HMGB1 was related to the poor prognosis of various tumours. HMGB1 is associated with and may be a potential marker in the prognosis of various tumours [28–30]. Studies have shown that HMGB1 is a chromatin component that is ubiquitous in mammalian cells. It has a variety of biological functions and plays an important role in cell migration, inflammation, cell differentiation and tumour metastasis. It has a role as a signal regulator of various biological functions such as the mediation of infection, injury and inflammation, promotion of autophagy, induction of cell death, and activation of natural immunity, playing a key role in the occurrence and development of tumors [46].

Logistic regression analysis showed that HPV infection and the severity of TCT were independent risk factors that could promote the occurrence of CIN, and low expression of ABCG2 and high expression of TDG and PCNA were risk factors that could promote the progression of CIN. ABCG2 is a member of the G subfamily of the ATP-binding cassette transporter superfamily. It is a multidrug resistance gene, also known as breast cancer resistance protein (BCRP), which has anti-tumour and anti-toxic effects [47]. Gupta et al. [23] used fluorescent quantitative PCR and immunohistochemistry to compare the expression of ABCG2 in normal and cervical cancer tissues, and they found low expression of ABCG2 in cervical cancer tissues, consistent with our results. Its down-regulation in tumours may play a role in tumour development by increasing tissue exposure to oncogene toxins and excessive production of nitric oxide. PCNA is the core protein in DNA synthesis, replication, and damage repair and a key factor in cell cycle regulation. It can be used as an indicator of tumour cell proliferation and tumour prognosis. Studies have shown that the expression of PCNA in cervical tissues increases with the increase in CIN and cervical cancer grade [24].TDG plays an important role in DNA demethylation [38]. But few studies have examined TDG in CIN and cervical cancer, so its role in the progression of CIN requires further analysis. It may be related to DNA methylation during the malignant transformation of cervical cells.

### Construction and screening of the early prediction model for evolution of CIN malignant transformation

According to the results of multivariate logistic regression analysis, risk factors that promote the occurrence and development of CIN, such as HPV positivity, TCT severity, ABCG2, PCNA and TDG genes, were used to establish random forest models with different combinations of these indicators. The results showed that model 4 (ABCG2 + PCNA+TDG) had the greatest accuracy and AUC value and the smallest OOB error value. Therefore, thecombination of ABCG2 + PCNA+TDG genes was selected as the best early prediction model for evolution of CIN malignant transformation [48].

## Summary

To our knowledge, this is the first study to use 16S rDNA sequencing to detect vaginal microbes at different levels of CIN and normal and cervical cancer tissues. The analysis suggests that with the severity of disease, the microbes show diversity, combined with high-risk HPV infection and disease levels. The relationship between vaginal microbial diversity may be related to the severity of high-risk HPV infection and CIN disease. This study is expected to clarify the microbiome changes related to the progression of CIN. Subsequently, we preliminarily screened out 10 candidate differential genes related to the progression of CIN in the cervical microbial community through bioinformatics analysis. Biological annotation and text mining of the relationships between genes, bacteria and CIN progress through the COREMINE database revealed a complex network relationship between candidate differential genes and the progress of bacteria and CIN. These 10 genes may be important candidate genes for community microbes to regulate CIN progression. Research based on these genes may provide new targets for blocking the progression of CIN. In the verification of these genes, we used the fluorescent quantitative PCR method to verify the expression of the candidate genes and found that ATM, ABCG2, PCNA, OGG1, TDG, LIG1, and HMGB1 were significantly expressed in three different cervical tissues. They might cooperate with HPV to participate in the occurrence and progression of CIN. Through ROC and logistic regression analyses, the factors associated with the occurrence and progression of CIN were initially screened out as HPV infection, TCT severity, HMGB1, ABCG2, and TDG. We used these indicators to establish a random forest model. Seven models were built through different combinations. The model 4 (ABCG2 + PCNA+TDG) had the highest accuracy and AUC value and the smallest OOB error value. Therefore, the combination of ABCG2 + PCNA+TDG genes was selected for the best early prediction model for evolution of CIN malignant transformation.

Current studies have found that the vaginal microecology is closely related to the occurrence and progression of CIN, but there has not been a study on the gene pathways contained in the flora or strains. This study firstly identified the genes from cervical microbial community that play an important role in the occurrence and progression of CIN. At the same time, the early warning model including ABCG2 + PCNA+TDG genes provided a new idea and target for clinical predictionand blocking the evolution of CIN malignant transformation from the aspect of cervical microbiological related genes. We comprehensively analyzed the risk factors for the occurrence and progression of CIN from clinical factors, microbial factors, and genetic levels. For the first time, we combined clinical factors and differential genes, and used the random forest model to select the best early warning model for the occurrence and progression of CIN. The purpose of this study is to find out the functional pathways of genes contained in the relevant flora and species related to the progress of CIN, to find the best early warning model for the occurrence and progression of CIN, and may provide an accurate clinical screening of CIN.

## Data Availability

The datasets generated and/or analysed during the current study are available in the NCBI repository, http://www.ncbi.nlm.nih.gov/bioproject/815961. Other datasets are available from the corresponding author on reasonable request.

## Abbreviations

CIN: Cervical intraepithelial neoplasia
PCR: Polymerase chain reaction
ROC: Receiver operating characteristic curve
HPV: Human papilloma virus
TCT: Thinprep cytology test
LSIL: Low-grade squamous intraepithelial lesion
HSIL: High-grade squamous intraepithelial lesion
ICC: Invasive cervical cancer
DNA: Deoxyribo Nucleic Acid
OTUs: Operational taxonomic units
AUC: Area under the curve of ROC
KEGG: Kyoto Encyclopaedia of Genes and Genomes database
FDR: False discovery rate
OD value: Optical density value
GWAS: Whole Transcriptome Association Study
BCRP: Breast cancer resistance protein
